# Oncologic Outcomes for Head and Neck Skin Malignancies Treated with Protons

**DOI:** 10.14338/IJPT-20-00045.1

**Published:** 2021-06-25

**Authors:** Jidapa Bridhikitti, Jason K. Viehman, W. Scott Harmsen, Adam C. Amundson, Satomi Shiraishi, Daniel W. Mundy, Jean-Claude M. Rwigema, Lisa A. McGee, Samir H. Patel, David M. Routman, Scott C. Lester, Michelle A. Neben-Wittich, Yolanda I. Garces, Daniel J. Ma, Robert L. Foote

**Affiliations:** 1 Prince of Songkla University, Songkhla, Thailand; 2 Division of Biomedical Statistics & Informatics, Mayo Clinic, Rochester, MN, USA; 3 Department of Biostatistics and Health Sciences Research, Mayo Clinic, Rochester, MN, USA; 4 Department of Radiation Oncology, Mayo Clinic, Rochester, MN, USA; 5 Department of Radiation Oncology, Mayo Clinic, Phoenix, AZ, USA

**Keywords:** skin cancer, intensity-modulated proton therapy, oncologic outcomes, adverse events

## Abstract

**Purpose:**

Radiation therapy (RT) is the standard treatment for patients with inoperable skin malignancies of the head and neck region (H&N), and as adjuvant treatment post surgery in patients at high risk for local or regional recurrence. This study reports clinical outcomes of intensity-modulated proton therapy (IMPT) for these malignancies.

**Materials and Methods:**

We retrospectively reviewed cases involving 47 patients with H&N malignancies of the skin (squamous cell, basal cell, melanoma, Merkel cell, angiosarcoma, other) who underwent IMPT for curative intent between July 2016 and July 2019. Overall survival was estimated via Kaplan-Meier analysis, and oncologic outcomes were reported as cumulative incidence with death as a competing risk.

**Results:**

The 2-year estimated local recurrence rate, regional recurrence rate, local regional recurrence rate, distant metastasis rate, and overall survival were 11.1% (95% confidence interval [CI], 4.1%-30.3%), 4.4% (95% CI, 1.1%-17.4%), 15.5% (95% CI, 7%-34.3%), 23.4% (95% CI, 5.8%-95.5%), and 87.2% (95% CI, 75.7%-100%), respectively. No patient was reported to have a grade 3 or higher adverse event during the last week of treatment or at the 3-month follow-up visit.

**Conclusion:**

IMPT is safe and effective in the treatment of skin malignancies of the H&N.

## Introduction

Surgical excision is the primary treatment for carcinomas and sarcomas of the skin [1–5]. However, radiation therapy (RT) is used in some situations, such as patients with disease in the head and neck region for whom surgery may result in cosmetic and/or functional deficits, patients with unresectable disease, or patients with multiple medical comorbidities. Radiation therapy also plays a role as an adjuvant treatment following surgery in patients with close or positive margins, satellite tumors, deep invasion, high-grade malignancies, bone or cartilage invasion, perineural invasion, lymph node metastases, extranodal extension, or recurrent disease.

There are several RT techniques for treating cutaneous lesions, including orthovoltage photons, 3-dimensional photon therapy, intensity-modulated photon radiation therapy (IMRT), electrons, and intensity-modulated proton radiation therapy (IMPT).

For scalp lesions, the dose distribution of electrons and orthovoltage RT is not conformal along the curved surfaces of the scalp. Large targets will require matched fields with these techniques, which are likely to introduce severe inhomogeneity. IMRT can conform dose to curved surfaces, resulting in increased homogenous tumor coverage as compared with other RT techniques [6–10]. However, the penetrating nature of megavoltage RT used by IMRT can lead to increased doses to the underlying brain [7–9] and eyes [7, 8] as compared with other RT techniques.

Ipsilateral neck RT in patients with skin lesions is indicated for treating primary lesions in the neck and for treating regional nodes, which requires exclusion of organs of the upper aerodigestive tract from the treatment volume. IMRT provides good dose conformity to the target volume. However, the integral dose to normal organs in the central or contralateral regions of the neck results in toxicity [11–14].

For larger lesions involving the face, IMRT is preferred because it can confine dose within the tumor target volume and restrict dose to the eyes, oral cavity, pharyngeal mucosa, and salivary glands. IMRT is also a recommended RT technique for tumors with cranial nerve invasion because nerve pathways to the skull, cavernous sinus, and brainstem are close to critical sensitive structures [15, 16]. However, IMRT has the limitation of entrance and exit dose in adjacent normal organs [17]. In some patients who have gross disease close to critical normal organs, it is extremely difficult to increase dose to a therapeutic level while keeping an acceptable dose constraint to the adjacent critical normal organs.

IMPT can produce conformal dose distributions around the tumor target volume like IMRT, but the distal dose is truncated, sparing adjacent structures from collateral dose. Therefore, it is expected in some situations that toxicities can be minimized by using IMPT for skin carcinomas and sarcomas in the head and neck region, compared with other RT modalities.

Previous studies of proton RT for treating patients with head and neck cancer, which included a small number of patients with skin malignancies, showed at least similar or better dose distributions in the tumor target volume [11, 18–22] with less dose to adjacent normal structures [11, 19–29] when compared to patients who were treated with IMRT. However, we are in need of data to confirm whether the dosimetric advantages from IMPT can result in clinical advantages.

To date, there are limited studies documenting the clinical outcome of patients with skin malignancies in the head and neck region treated with IMPT. The aim of this study is to provide oncologic outcomes of patients with skin carcinomas and sarcomas in the head and neck region treated with IMPT. A subsequent article will report patient-reported outcomes related to organ-at-risk dose-volume histogram statistics.

## Materials and Methods

### Study Design and Patient Population

This study is a retrospective study of skin carcinomas and sarcomas of the head and neck region treated with curative-intent IMPT between July 2016 and July 2019. The study was approved by the Institutional Review Board. Patients were excluded from analysis if they had previous RT to the same area, they did not give consent for their medical records to be used for research, they did not receive a complete course of IMPT, or they were treated with a mixture of photons and protons. Forty-seven patients were included. Demographic data, disease and treatment details, and patient status were extracted from the electronic health records.

### Treatment Details

#### Radiation therapy

All patients underwent computed tomography (CT) simulation with a customized head and neck rest, thermoplastic mask, and intraoral device for immobilization. Bolus helmets were used for proton energy degradation and optimizing beam spot size for superficial tumors that were close to critical organs, such as ocular structures. The relative biological equivalent of 1.1 was used for proton dose calculation. Commonly prescribed dose regimens are shown in Table 1.

**Table 1.** Commonly prescribed IMPT dose.

**Type of skin malignancy**	**Commonly prescribed IMPT dose (GyRBE 1.1)**	
	**Definitive IMPT**	**Adjuvant IMPT**
Squamous cell carcinoma, basal cell carcinoma, Merkel cell carcinoma, sarcoma	• Gross tumor 60-70 Gy, 2.0-2.5 Gy per fraction, 5 fractions per wk • Intermediate risk 63 Gy, 1.8 Gy per fraction SIB • Low risk and elective nodal regions 56-57 Gy, 1.6 Gy per fraction SIB	• Surgical bed 60-66 Gy, 2-2.2 Gy per fraction, 5 fractions per wk • Low risk and elective nodal regions 54 Gy, 1.8 Gy per fraction SIB
Melanoma	35 Gy, 7 Gy per fraction, 2 fractions per wk, Monday-Thursday or Tuesday-Friday	30 Gy, 6 Gy per fraction, 2 fractions per wk, Monday-Thursday or Tuesday-Friday

**Abbreviations:** IMPT, intensity-modulated proton therapy; SIB, simultaneous integrated boost.

For regions at intermediate or low risk of tumor extension, such as elective lymph nodes, a lower dose was prescribed. Stereotactic radiosurgery was used as a boost technique for intracranial gross tumor extension in 3 patients. Treatment volumes were determined and contoured by the treating radiation oncologist depending on the risk of microscopic tumor extension. If there were risks for cranial nerve involvement the nerves at risk were contoured retrogradely to the brain stem. Robust optimization was used with 3- to 5-mm translational setup uncertainties and 3% to 5% range uncertainties. The treatment planning system was Varian Medical System's Eclipse (Varian Medical Systems, Palo Alto, California).

IMPT planning used proton convolution superposition or an in-house Monte Carlo–based algorithm called Spock. Image-guided RT was performed daily by using 2D-3D kV imaging with 6-degrees-of-freedom couch adjustments with an option for in-room CT on rails volumetric verification. Weekly CT verification scans were performed on the in-room CT on rails with the patient in the treatment position, and replanning was performed when the target volume that received at least 95% of the prescribed dose (V95%) was < 95% or when a high-priority organ-at-risk dose deviation was > 5% or when a low-priority organ-at-risk dose deviation was > 10%.

#### Systemic therapy and surgery

All patients underwent multidisciplinary evaluation. Patients who were appropriate surgical candidates were treated with surgery. Most patients who received surgery at outside hospitals had their pathology specimens reviewed at Mayo Clinic and a surgeon was consulted if there was a concern for margin status. The aim of surgery for skin malignancies was to remove the tumor with negative margins and still maintain acceptable cosmesis, function, and quality of life. Systemic treatment was determined by the consulting medical oncologist. For 9 patients with squamous cell carcinoma, 5 were treated with concurrent cisplatin, 2 were treated with concurrent cemiplimab, 1 was treated with induction and concurrent cemiplimab, and 1 was treated with induction carboplatin, paclitaxel, and cetuximab and concurrent cisplatin. For 3 patients with melanoma, 1 was treated with adjuvant nivolumab following IMPT and 2 were treated with concurrent and adjuvant nivolumab.

### Adverse Events

Provider-reported adverse events related to IMPT were recorded prospectively during the last week of treatment and at the 3-month, 6-month, and 12-month follow-up visit by using Common Terminology Criteria for Adverse Events (CTCAE) version 4.03 (National Cancer Institute, Bethesda, Maryland).

### Statistical Methods

The events of interest were local recurrence, regional nodal recurrence, local regional recurrence, distant metastasis, and overall survival.

Patient characteristics, pathology, and dosimetry data are reported as median (interquartile range [IQR]) or percentage (N) as appropriate. Local recurrence, regional nodal recurrence, local regional recurrence, distant metastasis, and overall survival were calculated as the time from the last day of IMPT treatment to the date of the event or death. Recurrence and metastasis rates were estimated by using a competing risks model, and overall survival was estimated by using the Kaplan-Meier method. Effects of predictors were estimated by using Cox proportional hazards regression. Statistical significance was defined as *P* < .05. Statistical analyses were conducted with SAS version 9.4 (SAS Institute, Cary, North Carolina).

## Results

### Patient and Tumor Characteristics

Forty-seven patients were treated with IMPT. The median age was 72.8 years (IQR, 68.2-78.5). Eighty-five percent of patients were men. Sixty-eight percent of patients were diagnosed with squamous cell carcinoma. Other histologic types included melanoma, basal cell carcinoma, angiosarcoma, and Merkel cell carcinoma in 14.9%, 4.3%, 6.4%, and 2.1% of patients, respectively. The tumor location was in area M (cheek, forehead, scalp, and neck) in 62.5% of patients, and in area H (central face, eyelids, eyebrows, periorbital, nose, lips, chin, mandible, periauricular, and postauricular) in 37.5%. In approximately 50% of patients, primary and nodal classification could not be assessed. The details of patient and tumor characteristics are shown in Table 2.

**Table 2.** Patient and tumor characteristics.

**Characteristics**	**IMPT (n = 47)**
Age, median (IQR), y	72.8 (68.2-78.5)
Sex, % (n/total)	
Male	85.1 (40/47)
Female	14.9 (7/47)
Histologic type, % (n/total)	
Squamous cell carcinoma	68.1 (32/47)
Basal cell carcinoma	4.3 (2/47)
Melanoma	14.9 (7/47)
Merkel cell carcinoma	2.1 (1/47)
Angiosarcoma	6.4 (3/47)
Others^a^	4.3 (2/47)
Disease status (before IMPT), % (n/total)	
First diagnosis	53.2 (25/47)
Recurrent disease	46.8 (22/47)
Location of tumor, % (n/total)	
Area M^b^	62.5 (25/40)
Area H^c^	37.5 (15/40)
Missing	n = 7
Largest tumor size, median (IQR), cm	3 (2-4)
Depth of invasion, median (IQR), mm	12 (4-20)
T classification at initial diagnosis^d^, % (n/total)	
TX	48.9 (23/47)
T0/T1/T2	27.7 (13/47)
T3/T4	23.4 (11/47)
N classification at initial diagnosis^d^, % (n/total)	
NX	46.8 (22/47)
N0	34.0 (16/47)
N1/N2/N3	19.1 (9/47)
Perineural involvement, % (n/total)	
No	3.8 (1/26)
Yes	96.2 (25/26)
Missing	n = 21
Lymphatic involvement, % (n/total)	
No	50 (3/6)
Yes	50 (3/6)
Missing	n = 41
Vascular involvement, % (n/total)	
No	50 (3/6)
Yes	50 (3/6)
Missing	n = 41
Surgical margins, % (n/total)	
Negative (R0)	71.4 (25/35)
Positive (R1/R2)	28.6 (10/35)
Missing	n = 12

**Abbreviations:** IMPT, intensity-modulated proton therapy, IQR, interquartile range; R0, negative margins; R1, microscopically positive margins; R2, gross residual disease.

a Others: dermatofibrosarcoma protuberans, pleomorphic dermal sarcoma.

b Cheek, forehead, scalp, neck.

c Central face, eyelids, eyebrows, periorbital, nose, lips, chin, mandible, periauricular, postauricular.

d AJCC 8th edition,^30^ clinical and pathologic staging.

### Treatment Characteristics

Most patients were treated with surgery combined with IMPT with or without chemotherapy (surgery+IMPT, 59.6%; surgery+IMPT+chemotherapy, 14.9%). Chemotherapy was prescribed in 31.9% of patients. The median dose used was 60 Gy (IQR, 60-66). Three patients received a stereotactic radiosurgical boost for gross intracranial tumor extension with a median dose of 12 Gy (IQR, 10-12). The treatment volumes were grouped into ipsilateral neck (55.3%), scalp (25.5%), and face (17%). The median clinical target volume (CTV) for the high-dose region was 106 cm^3^ (IQR, 20-308). The median percentage of prescribed dose to 95% (D95%) of the high-risk CTV was 100% (IQR, 99%-101%). The details of treatment characteristics are shown in Table 3.

**Table 3.** Treatment characteristics.

**Characteristics**	**IMPT (n = 47)**
Treatment regimen, % (n/total)	
IMPT alone	8.5 (4/47)
Surgery+IMPT	59.6 (28/47)
IMPT+chemotherapy	17.0 (8/47)
Surgery+IMPT+ chemotherapy	14.9 (7/47)
IMPT indication, % (n/total)	
Definitive IMPT	27.7 (13/47)
Adjuvant IMPT	72.3 (34/47)
IMPT dose (GyRBE 1.1)	
Median (IQR)	60 (60-66)
Mean (SD)	57.7 (12.26)
IMPT region, % (n/total)	
Scalp^a^	25.5 (12/47)
Ipsilateral neck	55.3 (26/47)
Bilateral neck	2.1 (1/47)
Face^b^	17.0 (8/47)
IMPT volume, median (IQR), cm^3^	
High-dose CTV	106 (20-308)
Intermediate-dose CTV	280 (140-519)
Low-dose CTV	159 (46-528)
D95% high-dose CTV^c^ (%), median (IQR)	100 (99-101)

**Abbreviations:** IMPT, intensity-modulated proton therapy; IQR, interquartile range; CTV, clinical target volume.

a Forehead and scalp.

b Central face, eyelid, eyebrows, periorbital nose, lips, chin, mandible, cheek, periauricular, postauricular.

c Percentage of the prescribed dose that covered 95% of the CTV high-risk volume.

### Oncologic Outcomes

The median follow-up time was 1.31 years (IQR, 0.56-1.56). The details of disease recurrence and death are shown in Table 4.

**Table 4.** Disease recurrence and death.

**Event**	**IMPT 2-year estimates, % (95% CI)**
Local recurrence	11.1 (4.1-30.3)
Regional nodal recurrence	4.4 (1.1-17.4)
Local regional recurrence	15.5 (7-34.3)
Distant metastasis	23.4 (5.8-95.5)
Overall survival	87.2 (75.7-100)

**Abbreviation:** IMPT, intensity-modulated proton therapy; CI, confidence interval.

The 2-year estimated local recurrence rate was 11.1% (95% CI, 4.1%-30.3%). The 2-year estimated regional nodal recurrence rate was 4.4% (95% CI, 1.1%-17.4%). The 2-year death rate was 12.8% (95% CI, 0%-24.3%). The competing risks curves are shown in the Figure.

**Figure.**
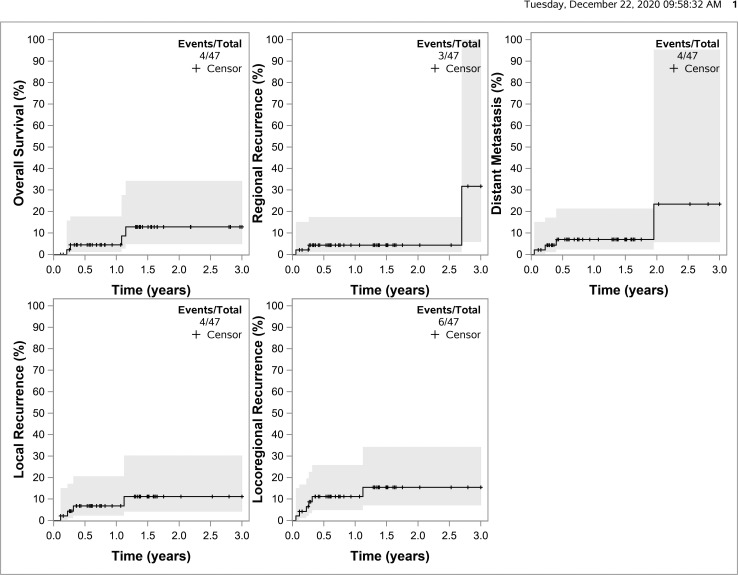
Competing risks curves for local recurrence, regional nodal recurrence, local regional recurrence, distant metastasis, and death.

In the largest squamous cell carcinoma histology subset, the 2-year estimated local recurrence, regional nodal recurrence, local regional recurrence, distant metastasis, and death rates were 17.3% (95% CI, 6.4%-46.7%), 3.1% (95% CI, 0.4%-22.2%), 20.0% (95% CI, 8.4%-47.9%), 23.3% (95% CI, 5.9%-92.2%), and 17.2% (95% CI, 0%-33.9%), respectively.

### Univariable Analysis

Univariable analysis revealed that treatment with surgery and postoperative adjuvant IMPT was associated with lower risk for local recurrence and local regional recurrence than IMPT alone. None of the other factors evaluated were found to be associated with local recurrence, regional nodal recurrence, local regional recurrence, distant metastasis, or death as shown in Table 5. A multivariable analysis was not performed owing to the small number of events.

**Table 5.** Hazard Ratio (95% Confidence Interval)

**Characteristic**	**Local recurrence (4 events)**	**Local regional recurrence (6 events)**	**Distant metastasis (4 events)**	**Overall survival (4 events)**
Age, per y	0.99 (0.92, 1.06)	0.98 (0.94, 1.03)	1.02 (0.92, 1.14)	1.10 (0.95, 1.27)
*P*	.70	.48	.70	.21
Histologic type				
Non–squamous cell	1.00 (reference)	1.00 (reference)	1.00 (reference)	1.00 (reference)
Squamous cell	4.96 (0.19, 131)	2.68 (0.33, 21.97)	1.07 (0.09, 12.31)	1.73 (0.18, 16.70)
*P*	.34	.36	.96	.64
Disease status				
First diagnosis	1.00 (reference)	1.00 (reference)	1.00 (reference)	1.00 (reference)
Recurrent disease	1.22 (0.18, 8.30)	2.55 (0.49, 13.21)	3.01 (0.32, 28.01)	3.77 (0.39, 36.31)
*P*	.84	.26	.33	.25
Location of tumor				
Area M^a^	1.00 (reference)	1.00 (reference)	1.00 (reference)	1.00 (reference)
Area H^b^	3.19 (0.30, 33.37)	1.05 (0.19, 5.87)	1.16 (0.20, 6.90)	0.46 (0.05, 4.47)
*P*	.33	.96	.87	.51
Largest tumor size, cm	0.60 (0.31, 1.18)	0.60 (0.35, 1.04)	0.82 (0.46, 1.47)	0.50 (0.13, 1.89)
*P*	.14	.07	.51	.31
T classification^c^				
Overall *P*	.73	.63	NA	NA
T0/T1/T2	1.00 (reference)	1.00 (reference)	NA	NA
TX	1.93 (0.18, 20.35)	2.54 (0.27, 23.74)	NA	NA
*P*	.59	.41	NA	NA
T3/T4	0.33 (0.01, 14.46)	1.21 (0.08, 19.64)	NA	NA
*P*	.80	.89	NA	NA
N classification^c^				
Overall *P*	NA	.56	.69	.84
N0	NA	1.00 (reference)	1.00 (reference)	1.00 (reference)
NX	NA	2.52 (0.25, 25.41)	4.32 (0.13, 143)	0.92 (0.13, 6.61)
*P*	NA	.43	.41	.93
N1/N2/N3	NA	3.68 (0.34, 39.60)	4.76 (0.11, 207)	0.35 (0.01, 12.39)
*P*	NA	.28	.42	.57
Surgical margins				
Negative	1.0 (reference)	NA	NA	1.0 (reference)
Positive	2.62 (0.18, 38.79)	NA	NA	0.76 (0.01, 74.54)
*P*	.48	NA	NA	.91
Treatment regimen				NA
Overall *P*	.14	.20	.33	
IMPT alone	1.00 (reference)	1.00 (reference)	1.00 (reference)	NA
Surgery+IMPT	0.07 (0.01, 0.76)	0.14 (0.02, 0.93)	0.13 (0.01, 1.71)	NA
*P*	.03	.04	.12	
IMPT+CT	0.07 (0.01, 2.77)	0.24 (0.03, 2.26)	0.09 (0.01, 4.94)	NA
*P*	.15	.21	.24	
Surgery+IMPT+CT	0.24 (0.03, 1.98)	0.24 (0.03, 2.00)	0.79 (0.08, 7.53)	NA
*P*	.18	.19	.84	
IMPT indication				
Definitive IMPT	1.00 (reference)	1.00 (reference)	1.00 (reference)	1.00 (reference)
Adjuvant IMPT	0.17 (0.02, 1.44)	0.19 (0.04, 1.03)	1.36 (0.13, 14.20)	0.14 (0.01, 1.32)
*P*	.10	.054	.80	.09

**Abbreviations:** NA, not analyzed; IMPT, intensity-modulated proton therapy; CT, chemotherapy.

a Area M: cheek, forehead, scalp, neck (reference).

b Area H: central face, eyelids, eyebrows, periorbital, nose, lips, chin, mandible, periauricular, postauricular.

c AJCC 8th edition,^30^ clinical and pathologic staging.

### Adverse Events

No patient was reported to have a grade 3 or higher adverse event during the last week of treatment or at the 3-month follow-up visit, including ocular toxicity, radiation dermatitis, dry mouth, dysgeusia, mucosal infection, oral pain, pharyngolaryngeal pain, salivary duct inflammation, brain necrosis, or osteoradionecrosis.

For grade 2 adverse events reported during the last week of treatment, 9 patients were reported to have radiation dermatitis, 3 dysgeusia, 3 oral pain, 2 pharyngolaryngeal pain, 2 dry mouth, 2 salivary duct inflammation, 2 ocular toxicity, and 1 mucosal infection. At the 3-month follow-up visit, 2 patients were reported to have grade 2 dry mouth, 1 skin ulceration with infection, and 1 dysgeusia.

## Discussion

This retrospective early report of skin malignancies of the head and neck region treated with IMPT suggests that IMPT is safe and effective in providing local and regional control of disease, particularly in the adjuvant setting for advanced disease.

There is limited research investigating proton therapy for skin malignancies of the head and neck region. One small retrospective trial from Japan studied the results of proton therapy in 6 patients with skin carcinomas. Good tumor response (at least 90% tumor regression in all patients) was observed [31]. Romesser et al [19] conducted a retrospective study in 41 patients with major salivary gland cancer or skin squamous cell carcinoma treated with ipsilateral neck proton or photon RT. Comparing passive scattering proton RT to IMRT, they reported no difference in tumor dose coverage or 1-year local regional tumor control.

There are several limitations in this retrospective study. The length of follow-up is relatively short. Longer follow-up will be required to document long-term oncologic outcomes and late adverse events. However, these early results are promising. Tumor and nodal classification could not be assessed in approximately 50% of patients. The reason for the missing initial staging information was the lack of this information in the medical record, which could not be determined by any means for patients referred from outside our institution. Another limitation is the variety of malignancies including squamous cell carcinoma, basal cell carcinoma, melanoma, Merkel cell carcinoma, angiosarcoma, and other malignancies, which may have differing disease control and survival outcomes. This is why we performed a separate analysis for the largest squamous cell carcinoma subgroup.

## Conclusion

IMPT is safe and effective in providing local and regional disease control for patients with skin malignancies of the head and neck region.
